# Does Time to Treatment Failure Measure the Same Clinical Process Across Oncology Facilities? Comment on Kajiura et al. Facility-Level Medical Oncology Specialist Availability and First-Line Time to Treatment Failure in Lung Cancer: A Nationwide C-CAT Registry Analysis. *Curr. Oncol.* 2026, *33*, 462

**DOI:** 10.3390/curroncol33100631

**Published:** 2026-10-20

**Authors:** Berkan Karabuga, Ekin Konca Karabuga, Osman Bilge Kaya

**Affiliations:** 1 Department of Medical Oncology, Dr. Abdurrahman Yurtaslan Ankara Oncology Training and Research Hospital, Ankara 06200, Türkiye; osmanbilge.kaya@saglik.gov.tr; 2 Department of Medical Oncology, Ankara Etlik City Hospital, Ankara 06170, Türkiye; ekin.koncakarabuga@saglik.gov.tr

## Abstract

Time to treatment failure (TTF) is a pragmatic endpoint that captures treatment discontinuation for multiple reasons. In a recent nationwide C-CAT registry analysis, Kajiura et al. examined whether facility-level medical oncology specialist availability was associated with first-line TTF among patients with lung cancer. We highlight two considerations that may influence the interpretation of facility-level differences in TTF: the heterogeneous reasons underlying treatment termination and the post–first-line selection inherent to C-CAT registration. Examining treatment-ending reasons and the timing of C-CAT registration across specialist-availability groups may help clarify whether differences in TTF reflect treatment duration itself or differences in cohort composition and treatment-discontinuation processes.

## 1. Introduction

We read with interest the study by Kajiura et al. examining the association between facility-level medical oncology specialist availability and first-line time to treatment failure (TTF) among patients with lung cancer in the nationwide C-CAT registry [1]. The authors should be commended for leveraging a national genomic registry to address an understudied health-services question. However, we believe that two interconnected features of the study warrant further consideration when interpreting facility-level differences in treatment duration: the heterogeneous composition of TTF and the post-first-line selection inherent to C-CAT registration.

## 2. Discussion

TTF is a pragmatic endpoint that captures treatment cessation for multiple reasons and has shown correlation with progression-free survival (PFS) in randomized oncology trials; however, the strength of this relationship varies according to treatment modality and clinical setting [2]. In the present study, recorded treatment termination included planned completion, toxicity, patient request, and a large category termed “invalidation cancellation,” which the authors appropriately did not equate with radiographic progression. Consequently, TTF events may represent fundamentally different clinical processes across patients. This heterogeneity becomes particularly relevant in a facility-level comparison, where treatment-discontinuation and documentation practices may themselves vary according to institutional organization and specialist availability. A longer TTF could therefore reflect prolonged disease control, but it could also result from different thresholds for treatment switching, toxicity management, planned treatment duration, or administrative recording. Importantly, if the relative contribution of these events differs between facility groups, the same TTF endpoint may not represent a comparable clinical construct across the groups being contrasted. We therefore wonder whether the reasons for treatment termination differed between facilities with 0–1 versus ≥2 JSMO specialists. A stratified description of treatment-ending reasons, followed where feasible by cause-specific analyses, could help determine whether the observed TTF differences reflect differences in treatment duration or differences in the processes leading to treatment discontinuation.

A second concern relates to cohort entry. As acknowledged by the authors, 99.3% of C-CAT registrations occurred after the initiation of first-line therapy. Although the original authors recognized potential sources of bias, we wish to further emphasize their implications for comparisons between facility groups. Thus, the analysis does not represent an inception cohort of patients starting first-line systemic treatment, but predominantly patients who survived and were subsequently selected or referred for comprehensive genomic profiling. Defining cohort inclusion based on processes occurring after treatment initiation can introduce time-related and selection biases in observational studies, particularly when the probability and timing of inclusion are related to prognosis or health-care processes [3,4,5]. This issue may be especially relevant here because specialist availability and facility infrastructure could themselves influence referral for genomic profiling and, consequently, entry into the C-CAT registry. Delayed-entry analyses appropriately account for the period before registry entry among patients who ultimately entered the cohort; however, they cannot account for patients who died, deteriorated, or were never referred for genomic profiling before C-CAT registration. If the likelihood of reaching C-CAT registration differed according to facility characteristics, the patients ultimately included in the two specialist-availability groups may therefore represent differently selected populations.

We therefore suggest that additional information on the interval between first-line treatment initiation and C-CAT registration according to specialist-availability group would strengthen the interpretation of the findings. Where data availability permits, stratifying the analysis by this interval, together with examining the distribution of treatment-ending reasons across specialist-availability groups, could help clarify whether observed TTF differences reflect treatment duration itself or differences in cohort composition and treatment-discontinuation processes. Importantly, the study demonstrates the potential of linking genomic registries with facility-level data; however, interpreting TTF as a facility-level treatment-process measure requires confidence that differences in both cohort entry and the composition of treatment-failure events do not systematically vary across the facilities being compared.

## Data Availability

No new data were created or analyzed in this study. Data sharing is not applicable to this article.

## Abbreviations

TTF	Time to treatment failure
PFS	Progression Free Survival
C-CAT	Center for Cancer Genomics and Advanced Therapeutics

## References

[1] Kajiura S., Satake H., Hayashi R., Yoshino T. (2026). Facility-Level Medical Oncology Specialist Availability and First-Line Time to Treatment Failure in Lung Cancer: A Nationwide C-CAT Registry Analysis. Curr. Oncol..

[2] Dalrymple J., Symons R., Sjoquist K.M., Lord S.J., Woodford R., Friedlander M., Simes R.J., Lee C.K. (2026). Time to Treatment Failure as an End Point in Pragmatic Oncology Trials: Association with Progression-Free Survival Across Randomized Studies. JCO Precis. Oncol..

[3] Suissa S. (2007). Immortal time bias in observational studies of drug effects. Pharmacoepidemiol. Drug Saf..

[4] Agarwal P., Moshier E., Ru M., Ohri N., Ennis R., Rosenzweig K., Mazumdar M. (2018). Immortal Time Bias in Observational Studies of Time-to-Event Outcomes: Assessing Effects of Postmastectomy Radiation Therapy Using the National Cancer Database. Cancer Control.

[5] Duchesneau E.D., Jackson B.E., Webster-Clark M., Lund J.L., Reeder-Hayes K.E., Nápoles A.M., Strassle P.D. (2022). The Timing, the Treatment, the Question: Comparison of Epidemiologic Approaches to Minimize Immortal Time Bias in Real-World Data Using a Surgical Oncology Example. Cancer Epidemiol. Biomark. Prev..

